# A Symmetry-Based Method to Infer Structural Brain Networks from Probabilistic Tractography Data

**DOI:** 10.3389/fninf.2016.00046

**Published:** 2016-11-04

**Authors:** Kamal Shadi, Saideh Bakhshi, David A. Gutman, Helen S. Mayberg, Constantine Dovrolis

**Affiliations:** ^1^School of Computer Science, Georgia Institute of TechnologyAtlanta, GA, USA; ^2^Department of Neurology, Psychiatry and Biomedical Informatics, Emory UniversityAtlanta, GA, USA; ^3^Psychiatric Neuroimaging and Therapeutics, Department of Psychiatry, Neurology, and Radiology, Emory UniversityAtlanta, GA, USA

**Keywords:** connectome, diffusion MRI, tractography, structural network, network analysis

## Abstract

Recent progress in diffusion MRI and tractography algorithms as well as the launch of the Human Connectome Project (HCP)^1^ have provided brain research with an abundance of structural connectivity data. In this work, we describe and evaluate a method that can infer the structural brain network that interconnects a given set of Regions of Interest (ROIs) from probabilistic tractography data. The proposed method, referred to as *Minimum Asymmetry Network Inference Algorithm (MANIA)*, does not determine the connectivity between two ROIs based on an arbitrary connectivity threshold. Instead, we exploit a basic limitation of the tractography process: the observed streamlines from a source to a target do not provide any information about the polarity of the underlying white matter, and so if there are some fibers connecting two voxels (or two ROIs) X and Y, tractography should be able in principle to follow this connection in both directions, from X to Y and from Y to X. We leverage this limitation to formulate the network inference process as an optimization problem that minimizes the (appropriately normalized) asymmetry of the observed network. We evaluate the proposed method using both the FiberCup dataset and based on a noise model that randomly corrupts the observed connectivity of synthetic networks. As a case-study, we apply MANIA on diffusion MRI data from 28 healthy subjects to infer the structural network between 18 corticolimbic ROIs that are associated with various neuropsychiatric conditions including depression, anxiety and addiction.

## Introduction

Diffusion MRI has opened a new window at the meso-scale structure of the living brain (Sporns et al., 2005). Clinicians and researchers can now observe and measure the properties of white matter in a non-invasive manner, analyzing the location and density of neuronal fibers at a spatial granularity of 1–2 mm isotropic voxels (Glasser et al., 2016b). Such structural information is important in deciphering how the brain works (Sporns, 2012, 2013), and it also creates new ways to understand and potentially diagnose (Ciccarelli et al., 2008; Fornito and Bullmore, 2015) or even treat (Mayberg et al., 2005) various brain diseases (Buckner et al., 2005; Bassett et al., 2008).

Processing diffusion MRI data using tractography algorithms is the next step forward: instead of analyzing the properties of white matter at the level of individual voxels, tractography aims to detect individual bundles of neuronal fibers originating or passing through a given “seed” voxel (Jbabdi et al., 2015). Additionally, given a seed voxel and a target ROI, it is now possible to examine the likelihood that some white matter fibers connect the two (referred to as “probabilistic tractography”), and to track the shape of these connections (Behrens et al., 2007). In this paper, we propose a method to further process the noisy connectivity information provided by probabilistic tractography in order to estimate an interconnection network between a given set of gray matter ROIs.

Diffusion MRI data, jointly with deterministic (Mori et al., 1999) or probabilistic tractography methods (Behrens et al., 2007) have been used successfully during the last decade to infer the structure of the human brain between hundreds of ROIs (Hagmann et al., 2007). Various structural properties of these networks have been discovered for the healthy brain (Bassett et al., 2011) and for various psychiatric diseases (McIntosh et al., 2008; Daianu et al., 2013). When combined with fMRI and behavioral or genomic analysis, these non-trivial topological properties provide new insights about the role of individual ROIs in specific networks and the way in which these distinct ROIs exchange information to produce integrated function (Damoiseaux and Greicius, 2009; Greicius et al., 2009).

A major challenge in this research effort is that the inferred brain networks, as well as their topological properties, are often sensitive to the parameters of the tractography process (Rubinov and Sporns, 2010; Bastiani et al., 2012; Duda et al., 2014; Thomas et al., 2014). In probabilistic tractography, the most critical of those parameters is the *connectivity threshold* τ that determines whether the tractography-generated streamlines from a given seed voxel to a target ROI occur with sufficiently large probability to indicate the presence of an actual connection (Li et al., 2012b). If τ is too low the resulting network includes connections that do not exist in reality and the converse happens if τ is too high. Even a small number of spurious or miss-detected edges can adversly effect the properties of the inferred networks (Van Wijk et al., 2010). Further, the optimal value of τ, i.e., the threshold that would result in the most accurate reconstruction of the underlying “ground truth” network, may vary between different subjects (Gong et al., 2009) and image acquisions parameters (Jones et al., 2013).

The problem of selecting an appropriate connectivity threshold in either deterministic or probabilistic tractography is not new. One approach has been to select the largest possible threshold (i.e., fewest possible edges) so that the final inferred network remains connected across the majority of subjects (Li et al., 2009). Depending on the selected ROIs, this approach can lead to many miss-detections (if those ROIs are densely interconnected) or false alarms (if the ROIs are not directly connected).

Another approach has been to analyze the tractography data with a wide range of threshold values, hoping that certain qualitative properties are robust and independent of the exact threshold. Li et al. investigated how the connectivity thresholds affects network density and therefore network efficiency metrics (Li et al., 2012b). Duda et al. have shown the importance of the connectivity threshold for various network metrics such as clustering coefficient and characteristic path length (Duda et al., 2014).

This work focuses on the following problem: how to infer the structural network between a given set of gray matter ROIs in a reliable way that does not require an arbitrary choice of the connectivity threshold? The proposed method, referred to as Minimum Asymmetry Network Inference Algorithm (MANIA), exploits a fundamental limitation of diffusion MRI imaging and of the tractography process: diffusion MRI can estimate the orientation of fibers in each voxel but it cannot infer the polarity (afferent vs. efferent) of those fibers (Robinson et al., 2010; Jbabdi and Johansen-Berg, 2011). Similarly, a tractography algorithm can combine those per-voxel orientations to “stitch together” expected connections but it does not provide any information about the direction of those connections (Hagmann et al., 2008; Donahue et al., 2016). Given this limitation, MANIA expects that the presence of an actual connection from voxel X to voxel Y (in that direction) will be detected by the tractography process as a symmetric connection between X and Y. Similarly, if there is no connection between X and Y, the tractography process should not detect a connection in either direction. Based on this principle, MANIA formulates the network inference problem as an optimization over the range of connectivity threshold values: it selects the value of τ that minimizes the asymmetry of the resulting network. The network asymmetry is normalized relative to the asymmetry that would be expected due to chance alone in a random network of the same density.

MANIA can work in tandem with all probabilistic tractography methods, such as FSL's probtrackx (Behrens et al., 2007), PiCo (Parker et al., 2003), and fDF-PROBA (Descoteaux et al., 2009). It can be also combined with deterministic tractography methods, such as FACT (Mori et al., 1999), but only if a large number of streamlines (in the thousands) are generated from randomly placed seeds within each voxel (Cheng et al., 2012).

We expect that the given set of ROIs primarily reside in gray matter. Dilating a gray matter ROI so that it includes some white matter voxels may result in connectivity errors, especially with cortical ROIs, because of the dense white matter systems just beneath the cortical sheet (Reveley et al., 2015). The selection of ROIs and the estimation of their boundaries is an important issue that is further discussed in the Discussion.

We evaluate the accuracy of MANIA using both the well-known FiberCup dataset and based on synthetically generated data in which the ground-truth network is known. We also compare MANIA with an ideal threshold-based method in which the optimal connectivity threshold is assumed to be known. Further, we show how to associate a confidence level with each edge, and how to apply MANIA in a group of subjects. Finally, as a case-study, we apply MANIA on diffusion MRI data from 28 healthy subjects (Chen et al., 2013) to infer the structural network between 18 corticolimbic ROIs that are implicated with neuropsychiatric disorders such as major depressive disorder (MDD), post-traumatic stress disorder (PTSD), obsessive compulsive disorder (OCD), generalized anxiety disorder (GAD) and addictive disorder (AD) (Seminowicz et al., 2004; Elman et al., 2013; Beucke et al., 2014; Peterson et al., 2014). We note however that, even though these ROIs are generally associated with various psychiatric disorders and the aspects of emotional regulation putatively impacted by these disorders, the objective of this work is *not* to infer the network that is associated with any particular disorder.

## Materials and methods

### MANIA inputs

**Figure 1:**
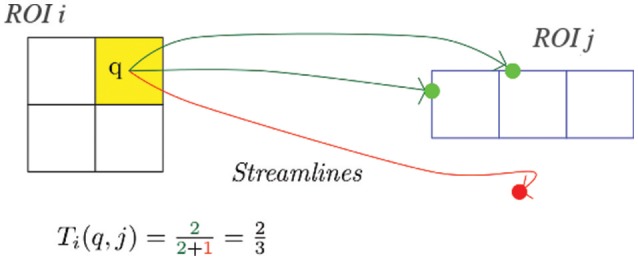
**Running tractography with streamlines from a seed voxel ***q*** in the ***i***'th ROI to the ***j***'th target ROI**. Two streamlines hit the target ROI, therefore $T_{i}\left(q,\;j\right)=\frac{2}{3}$.

The proposed network inference method requires the following inputs:

1. A set of *N* ROIs that represent the nodes of the structural brain network. The *i*'th ROI is a spatially connected cluster of *v*_*i*_ voxels (*i* = 1⋯*N*). The selection of ROIs is important (Zalesky et al., 2010; de Reus and van den Heuvel, 2013b) but outside the scope of MANIA. MANIA attempts to find the anatomic network between the given ROIs independent of whether the latter are defined by an expert neuroanatomist or by a data-driven method. For instance, ROIs may correspond to different Brodmann areas or other anatomical atlases (Tzourio et al., 1997; Petrides, 2005). Or, it could be that the spatial extent of ROIs results from the analysis of fMRI data (McKeown et al., 1997; Craddock et al., 2012; Blumensath et al., 2013; Thirion et al., 2014). ROIs can also be defined based on both fMRI analysis and anatomical knowledge (Glasser et al., 2016a).
2. The results of the tractography process between the previous *N* ROIs. We assume that the tractography results are structured as voxel-to-ROI matrices (i.e., streamlines are generated from each voxel toward each target ROI), instead of voxel-to-voxel or ROI-to-ROI matrices. Specifically, we represent the output of tractography with *N* matrices *T*_*i*_(*i* = 1⋯*N*), defined as follows. The *i*'th ROI corresponds to a matrix *T*_*i*_ with *v*_*i*_ rows (i.e., the number of voxels in that ROI) and *N* columns. The element (*j, k*) of matrix *T*_*i*_ represents *the fraction of tractography-generated streamlines that originate from the seed voxel j* of the *i*'th ROI *and reach any voxel of the k*'th *ROI*. The same number of streamlines is generated for every seed to target pair (*j, k*). The *i*'th column of matrix *T*_*i*_ is set to zero, meaning that we do not consider edges from an ROI back to itself (even if such fibers exist). Figure 1 illustrates this notation. Since we have *N* ROIs, there will be *N* input matrices, one for each source ROI.

### Connectivity threshold τ

How can we decide whether voxel *j* of the *i*'th ROI connects to the *k*'th ROI given the fraction *T*_*i*_(*j, k*)? The simplest approach is to examine if *T*_*i*_(*j, k*) is larger than a given “*connectivity threshold”* τ (0 < τ < 1). In MANIA, τ is not a given threshold but an optimization variable, as described in the next section.

As in prior work, we assume that the *i*'th ROI is connected to the *k*'th ROI as long as at least one voxel of the former is connected to the latter (Hagmann et al., 2008; Bassett et al., 2011). This assumption is not central to MANIA however, and it can be easily replaced with a stronger connectivity constraint, depending on the size of the given ROIs and the spatial resolution at which those ROIs are specified.

### Network inference as an optimization problem

For a given value of τ, we can identify the voxels of the *i*'th ROI that connect to every other ROI. If this process is repeated for every *i*, we can construct a directed network in which the *i*'th ROI is connected to the *j*'th ROI if there is at least one voxel in the former that is connected to the latter for that value of τ. This network can be represented with an adjacency matrix^2^ *G*_τ_, as follows:

(1) $$G_{\tau }\left(i,k\right)=\;\{\begin{array}{cc} 1 & if\;T_{i}\left(j,k\right)>\tau \;for\;at\;least\;one\;voxel\;j\\ 0 & otherwise \end{array}$$

So, the element (*i, k*) of this matrix is equal to one if there is a (directed) edge from the *i*'th ROI to the *k*'th ROI. The diagonal entries of *G*_τ_ are set to zero because we do not consider streamlines from a source ROI back to itself.

We define the *asymmetry* ϕ(*G*) of a directed network *G* as the fraction of edges that are present in only one direction,

(2) $$\begin{array}{l} \phi \left(G\right)\;=\;\frac{\sum_{i=1}^{N}\sum_{k=1}^{N}G\left(i,k\right)\left(1-G\left(k,i\right)\right)}{\sum_{i=1}^{i=N}\sum_{k=1}^{N}G\left(i,k\right)} \end{array}$$

The asymmetry of a network *G* depends on its *density* ρ (*G*), defined as the fraction of connected node-pairs,

(3) $$\begin{array}{l} \rho \left(G\right)=\frac{\sum_{i=1}^{N}\sum_{k=1}^{N}G\left(i,k\right)}{N\left(N-1\right)} \end{array}$$

The more edges a directed network has, the more likely it becomes that a pair of nodes will be connected in both directions, i.e., the higher the density, the lower the asymmetry.

More formally, consider a directed network with K directed edges and N nodes. The density is ρ = *K*/[*N*(*N* − 1)] (0 < ρ <1). To construct such a network randomly, denoted by *G*_ρ_, we simply connect *N*(*N* − 1)ρ randomly selected but distinct pairs of nodes with directed edges. The expected number of edges that exist in only one direction is *N*(*N* − 1)ρ (1 − ρ) and so the expected value of the asymmetry of *G*_ρ_ is:

(4) $$\begin{array}{l} \bar{\phi }\left(G_{\rho }\right)=\frac{N\left(N-1\right)\rho \left(1-\rho \right)}{N\left(N-1\right)\rho }=1-\rho \end{array}$$

To quantify the actual asymmetry of an observed network *G*_τ_, we normalize ϕ(*G*_τ_) by the asymmetry that is expected simply due to chance given the density of this network. So, we define the *normalized asymmetry* of *G*_τ_ as

(5) $$\begin{array}{l} \Phi \left(G_{\tau }\right)=\frac{\phi \left(G_{\tau }\right)}{\bar{\phi }\left(G_{\tau }\right)} \end{array}$$

which is well defined as long as ρ (*G*_τ_) <1.

MANIA is based on the following premise: *the inferred directed network should be as symmetric as possible*. The reason is that the tractography process is unable to infer the actual direction (polarity) of the underlying neural fibers. So, if there are some fibers connecting two voxels X and Y, tractography should be able, in principle, to follow this connection in both directions, from X to Y and from Y to X. We do not claim that two connected ROIs are always attached with both afferent and efferent fibers; instead, we argue that tractography is not able to discover the polarity of those fibers and so the corresponding connection should be trace-able in both directions.

The presence of some streamlines from some voxels in ROI X to ROI Y does not necessarily mean however that the network inference method will detect an edge both from X to Y and from Y to X; this also depends on the parameter τ. Given that we aim to minimize the asymmetry of the inferred network, MANIA aims to select the value of τ that leads to the lowest possible asymmetry.

The corresponding optimization problem can be stated as follows: determine the adjacency matrix $\;\hat{G}\;=\;G_{\tau }^{*}$, where τ^*^ is a value of the connectivity threshold that minimizes the normalized asymmetry of *G*_τ_ across all possible values of τ,

(6) $$\begin{array}{l} \tau^{*}\;=\;\arg{min}_{0<\tau <1}\;\Phi \left(G_{\tau }\right) \end{array}$$

So, MANIA is based on the premise that there is an ideal value (or range of values) of the connectivity threshold that can correctly classify every directed pair of ROIs as either “connection exists” or “connection does not exist.” When such a threshold exists, it will result in a completely symmetric network (because a perfectly accurate tractography-based network cannot be asymmetric). On the other hand, if such an ideal threshold does not exist (for instance, it may be that two connected ROIs are too far from each other and tractography cannot “see” their connection, or that it is impossible for streamlines to cross the White matter/Gray matter Boundary (WGB) of a certain ROI in one direction but not in the opposite), then MANIA aims to at least minimize the normalized asymmetry metric, even if the resulting network will not be completely symmetric.

If there is more than one value of τ that results in the same minimum of the normalized asymmetry (potentially zero), MANIA reports the network with the largest density. The rationale behind this tie-breaker is to avoid trivial solutions that include only a subset of the actual network edges. The previous optimization problem can be solved numerically by scanning the range of τ values with a given resolution^3^. The density of the resulting network is denoted by

(7) $$\begin{array}{l} \rho^{*}\;=\;\rho \left(G_{\tau^{*}}\right) \end{array}$$

**Figure 2:**
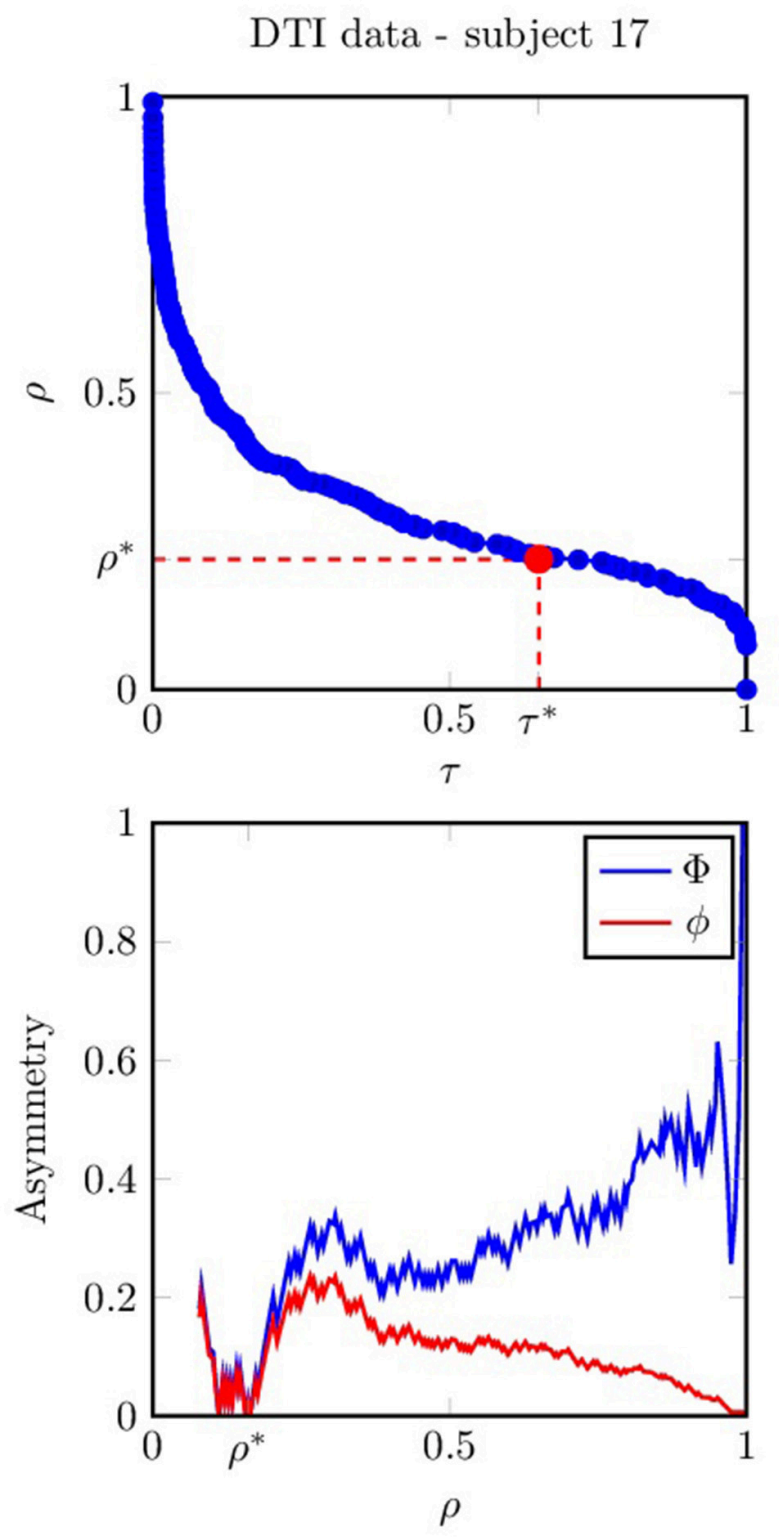
**(Top)** Network density ρ as a function of the connectivity threshold τ (plotted for one subject in our DTI dataset). **(Bottom)** Network asymmetry (red) and normalized network asymmetry (blue) as functions of the network density ρ. The optimal density ρ^*^ is the largest value that minimizes the normalized network asymmetry.

As an illustration of the previous method, Figure 2 shows how the network asymmetry (both ϕ and Φ) varies with the density ρ as well as the relation between ρ and τ for the dataset that corresponds to one of the subjects in our case-study.

### Threshold-based network inference with post-symmetrization

A common network inference method is to rely on a given connectivity threshold τ, as shown in Equation (1). This threshold is sometimes chosen to achieve a certain network density or to ensure that the network is connected (Li et al., 2009).

Given that tractography cannot detect the direction of inferred edges, the resulting network is often “post-symmetrized” (Azadbakht et al., 2015; Donahue et al., 2016). Consider two network nodes *i* and *j* and suppose that the fraction of streamlines from *i* to *j* is denoted by *T*_*i, j*_. Supose that *T*_*i, j*_ > τ but *T*_*j, i*_ < τ, where τ is a given threshold (not necessarily the threshold chosen by MANIA). We can resolve this conflicting evidence between the two directions of the edge by comparing the ratio (*T*_*i, j*_ − τ)/(1 − τ), which reflects our confidence that the edge from *i* to *j* exists, with the ratio (τ − *T*_*j, i*_)/τ that reflects our confidence that the edge from *j* to *i* does *not* exist. The two nodes are connected with an (undirected) edge only if the former ratio is larger.

If MANIA cannot find a threshold value that gives a completely symmetric network, the aforementioned post-symmetrization method is applied on the network that results from MANIA.

### Performance metrics

MANIA can be viewed as a binary classifier: each possible directed edge is classified as *present* or *absent*. We evaluate MANIA based on the following standard metrics for binary classification: the *false positive rate* (or false alarm) *p*_*f*_, and the *false negative rate* (or miss detection) *p*_*m*_. The former is defined as the fraction of absent edges that are incorrectly classified as present, while the latter is defined as the fraction of present edges that are incorrectly classified as absent.

Also, the *Jaccard similarity* between the sets of edges *E*(*G*) and *E*(Ĝ) of the actual network *G* and the MANIA network Ĝ, respectively, is defined as

(8) $$\begin{array}{l} J\left(G,Ĝ\right)=\frac{\left|E\left(G\right)\cap E\left(Ĝ\right)\right|}{\left|E\left(G\right)\cup E\left(Ĝ\right)\right|} \end{array}$$

and it varies between zero (no common edges) and one (identical networks).

### Optimal threshold-based network inference

We can also compare the network that results from MANIA with the network that would result if we knew the optimal value τ^*opt*^ of the connectivity threshold, i.e., the value of τ that maximizes the Jaccard similarity between the inferred network *G*_τ_ and the ground truth network *G*:

(9) $$\begin{array}{l} \tau^{opt}=\arg max_{0<\tau <1}\;J\left(G_{\tau },G\right) \end{array}$$

Even though it is not possible to know this optimal threshold value when analyzing real tractography data, we can easily compute its value (or range of values) in experiments with synthetically generated networks, where the ground-truth network *G* is known.

### Edge ranking and confidence metric in MANIA

The output of MANIA is an unweighted directed network. We can quantify the level of confidence we have in each edge with the following edge ranking scheme.

**Figure 3:**
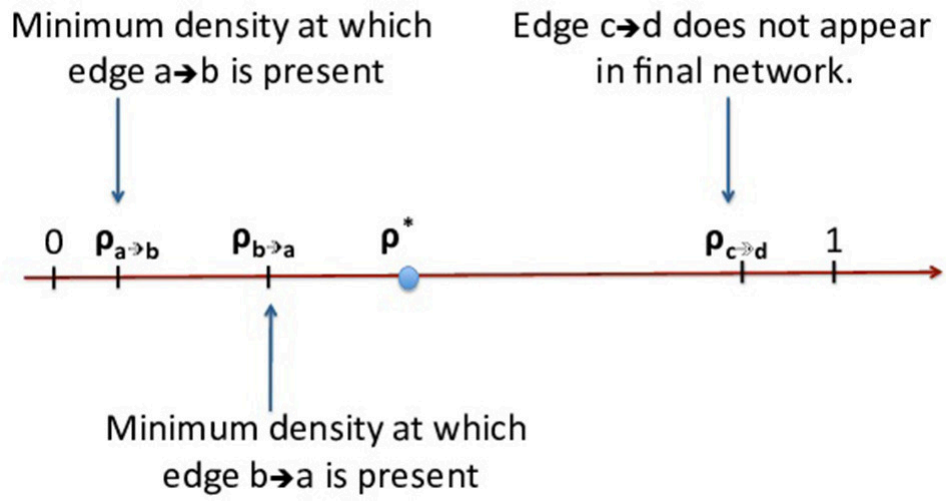
**As we decrease the connectivity threshold, each edge first appears at a certain value of the network density**. If this density is larger than ρ^*^, the corresponding edge is *not* present in the MANIA network.

Let ρ_α_ be the minimum network density at which edge α is present. If the edge α is from a source X to a target Y, the lower ρ_α_ is, the higher the fraction of streamlines from X to Y. Consequently, we can rank edges so that we are more confident in the presence of edge α than of edge β if ρ_α_ < ρ_β_ (see Figure 3).

We define a *confidence metric* for an edge α that is present in the MANIA network (i.e., $\rho_{\alpha }<\rho^{*}$) as follows

(10) $$\begin{array}{l} C\left(\alpha \right)=\frac{\rho^{*}-\rho_{\alpha }}{\rho^{*}} \end{array}$$

*C* (α) varies from 0 (the edge is only marginally present) to 1 (highest confidence that the edge is present). Similarly, if edge α is absent from the MANIA network (i.e., $\rho_{\alpha }>\rho^{*}$), its confidence metric is defined as

(11) $$\begin{array}{l} C\left(\alpha \right)=\frac{\rho^{*}-\rho_{\alpha }}{1-\rho^{*}} \end{array}$$

and *C* (α) varies from 0 (the edge is only marginally absent) to −1 (highest confidence that the edge is absent).

We also define a confidence metric for a pair of nodes (*X,Y*), as the arithmetic mean of the confidence metric of the two directed edges between *X* and *Y*,

(12) $$\begin{array}{l} C\left(X,Y\right)=\frac{C\left(X\to Y\right)+C\left(Y\to X\right)}{2} \end{array}$$

Note that one of the two edges may be present while the other may be absent. In that case, the confidence of the corresponding node-pair will be less than the confidence of the present edge.

Note that this edge confidence metric is not related to connection “strength” or “quality,” and the resulting network is still meant to be interpreted as an unweighted graph.

### Group analysis using MANIA

If the objective is to create a single “average network” based on data from several subjects, the question is how to best aggregate the tractography data from the given group. One approach is to average the diffusion MRI data, after transforming them in a standard space. Another approach is to average the fraction of streamlines from a given seed voxel to a given target ROI, across all subjects. The previous approaches can be sensitive to outliers, variations in the diffusion MRI process across subjects, tractography errors and mapping/warping into a standard space. A recently proposed method is to construct a group-level network based on the consistency of the edge weights across subjects (Roberts et al., 2016); this approach preserves edges that appear consistently strong for their length, rather than penalizing long-distance connections. This approach may not be applicable however when the group size is small.

Another method is to construct an individual network for each subject, perhaps using MANIA, and then form an aggregate network by only keeping those edges that appear in a large fraction of subjects. This approach requires a group-level threshold for the minimum fraction of subjects that should have a connection. For instance, de Reus et al. have proposed a statistically rigorous method to compute such a threshold (de Reus and van den Heuvel, 2013a).

Here we propose a different group analysis method, referred to as *group-MANIA*, that is based on the aggregation of edge-rankings across subjects. The rank-based nature of this method makes it robust to outliers.

As in the previous section, the edges of a subject can be ranked based on the minimum network density at which an edge first appears. We are more confident in the presence of edge α than of edge β if ρ_α_ < ρ_β_ (see Figure 3).

Suppose that we compute an edge rank vector *R*_*m*_ for each subject *m*, so that the lowest rank *R*_*m*_(1) corresponds to the edge for which we are most confident. The number of possible (not necessarily present) directed edges is the same for all subjects: *N* (*N* − 1) where *N* is the number of network nodes.

Given a group of size *M*, we have *M* distinct rank vectors *R*_*m*_, *m* = 1⋯*M*. The computational problem of *rank aggregation* (Schalekamp and van Zuylen, 2009) is to compute an optimal permutation $\hat{R}$ of the *N* (*N* − 1) possible edges that captures as well as possible the ordering relations in the *M* input rank vectors. Specifically, the *Kemeny distance* between two rank vectors *R*_1_ and *R*_2_ is defined as

(13) $$\sum_{i}\sum_{j}\delta_{i,j}(R_{1},R_{2})$$

where δ_*i, j*_(*R*_1_, *R*_2_) = 1 if *R*_1_ and *R*_2_ disagree in the relative position of elements *i* and *j*, and zero otherwise. Rank aggregation aims to compute a vector $\hat{R}$ that minimizes the cumulative Kemeny distance between $\hat{R}$ and all input rank vectors *R*_*m*_. It is an NP-hard problem, and so it is typically solved heuristically. We use the Quicksort algorithm (Ailon et al., 2008) since it is has been shown to provide a good approximation of the optimum solution. QuickSort selects a random edge as pivot at each recursive step, while the remaining edges are separated in a left and right list. The left list includes edges that have a lower rank than the pivot in the majority of the subjects; similarly for the right list. The algorithm proceeds recursively in the left and right lists until all edges are ordered.

After computing the optimal aggregate rank vector, we apply MANIA on $\hat{R}$ to compute the network with the minimum normalized asymmetry (as in the case of a single subject). Note however that the input to MANIA in this case is an ordered list of edges $\hat{R}$ rather than the set of connectivity matrices *T* (see the MANIA Inputs section). Group-MANIA forms a network with the first *K* = *N*(*N* − 1)ρ edges in $\hat{R}$, and it computes the normalized asymmetry of that network. It then repeats this step, for all values of K, to identify the network with the minimum value of Φ. We refer to the resulting network as the *rank-aggregated network*.

### Fibercup phantom dataset

We utilize the publicly available FiberCup dataset (Poupon et al., 2008, 2010; Fillard et al., 2011) as one of the benchmarks to evaluate MANIA's accuracy. We use a FiberCup acquisition in which the *b*-value is 1500. FiberCup resembles a human brain coronal section with 18 ROIs and seven fiber bundles—see Figure 2 of Neher et al. (2014). These fiber bundles are constructed to include sharp turns as well as crossing, kissing and branching points, making it a challenging benchmark for any tractography algorithm. Due to the practical difficulties of generating a realistic phantom, FiberCup does not contain any complex 3D fiber structures.

We apply probtrackx and MANIA on the FiberCup phantom to infer the network of the seven underlying connections. The FDT probabilistic tractography parameters are set to their default values (number of streamlines = 5000, maximum number of steps = 2000, loop check: set, curvature threshold = 0.2, step length = 0.5 mm, no distance bias correction). Probtrackx is seeded at the WGB of the ROIs that are connected to fiber terminals.

### Synthetically generated networks

To evaluate the accuracy and sensitivity of MANIA in a reliable manner we need to rely on synthetic networks rather than actual DTI and tractography data. The benefit of these computational experiments is that we can test MANIA under a wide range of noise conditions and for arbitrary network densities. Unfortunately there are no good statistical models for the noise in the output of tractography data (Jbabdi and Johansen-Berg, 2011). We evaluate MANIA based on a simple noise model that is based on the theory of *maximum entropy distributions*, as described next.

For simplicity, each ROI of the synthetically generated networks is simply a voxel. Modeling multi-voxel ROIs in these simulation experiments would not add any new insights. Suppose that the directed network between *N* nodes is represented by the *N* × *N* adjacency matrix *G*. Let *T*_*i, j*_ be the fraction of streamlines that originate from node *i* and terminate at node *j*. Ideally, in the absence of any noise in the DTI data and without any errors in the tractography process, it should be that

(14) $$T_{i,\;j}=\;\left\{ \begin{array}{l} 1\;if\;G_{i,\;j}=1\;or\;G_{j,\;i}=1\\ 0\;if\;G_{i,\;j}=0\;and\;G_{j,\;i}=0 \end{array}\right.$$

So, if there is an edge between nodes *i* and *j* in either direction, the fraction of streamlines from node *i* to node *j* should be 100%; otherwise, it should be zero.

In practice, there is significant noise in DTI data and the tractography process can be error-prone, especially when the ROIs are in gray matter and/or when neural fibers cross each other, split or merge, or fan out as they approach their targets. Consequently, the tractography output may show that some streamlines do not reach from node *i* to node *j* even when the two nodes are connected, or that some streamlines get from *i* to *j* even when there is no connection between the two nodes. We model these errors probabilistically, as follows:

(15) $$T_{i,\;j}=\{\begin{array}{ll} 1-Z_{1} & \;if\;G_{i,\;j}=1\;or\;G_{j,\;i}=1\\ Z_{2} & \;if\;G_{i,\;j}=0\;and\;G_{j,\;i}=0 \end{array}$$

where *Z*_1_ and *Z*_2_ are two (generally different) random variables with [0, 1] support. If their probability mass is concentrated close to 0, the results of the tractography process are not significantly affected by noise. On the other hand, if these two random variables are uniformly distributed in [0, 1], the tractography results are completely random and any network inference process is hopeless.

We model the two random variables *Z*_1_ and *Z*_2_ with the *Maximum Entropy distribution with one-degree of freedom*. In this case, this distribution is the truncated exponential distribution with support [0,1],

(16) $$f_{Z}(\zeta )=\{\begin{array}{ll} e\frac{\alpha }{1-e^{-\alpha }}e^{-\alpha \zeta }\;\zeta & \in [0,1]\\ 0 & otherwise,\; \end{array}$$

where α > 0 is a parameter that determines the mean and variance of the distribution. Instead of controlling α, we control the intensity of noise through the mean of *Z*,

(17) $$\begin{array}{l} \mu =E\left[Z\right]=\frac{1-\left(1+\alpha \right)e^{-\alpha }}{\alpha \left(1-e^{-\alpha }\right)} \end{array}$$

**Figure 4:**
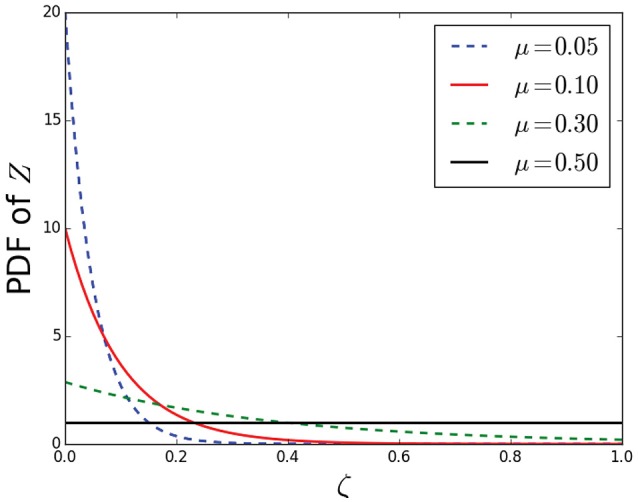
**Probabilistic error model (Z) for tractography-generated connection probabilities using the maximum entropy distribution (with one degree of freedom)**.

The two distributions *Z*_1_ and *Z*_2_ follow this statistical model with means μ_1_ and μ_2_, respectively. Figure 4 shows the previous distribution for four values of μ. Note that the distribution *Z* becomes almost “flat” (close to the uniform distribution) when its mean is higher than 0.3, meaning that tractography would be extremely inaccurate when the noise intensity exceeds that level. In the rest of this paper we limit the range of μ_1_ and μ_2_ between 0 and 0.3.

### DTI data, tractography parameters, and selected ROIs

We also apply MANIA in Diffusion Tensor Imaging (DTI) data collected by an earlier study: “Brain aging in humans, chimpanzees (Pan troglodytes), and rhesus macaques (Macaca mulatta): magnetic resonance imaging studies of macro- and microstructural changes” (Chen et al., 2013). All subjects gave written informed consent, and the study was approved by the Emory University Institutional Review Board. We analyzed the data for 28 of those subjects mostly based on handedness (right-handed females, without a history of psychiatric disorder). Diffusion-weighted images were acquired using a Siemens 3T with a 12-channel parallel imaging phase-array coil. Foam cushions were used to minimize head motion. Diffusion MRI data were collected with a diffusion weighted SE-EPI sequence (Generalized Autocalibrating Partially Parallel Acquisitions [GRAPPA] factor of 2). A dual spin-echo technique combined with bipolar gradients was used to minimize eddy-current effects. The parameters used for diffusion data acquisition were as follows: diffusion-weighting gradients applied in 60 directions with a *b*-value of 1000 s/mm^2^; TR/TE of 8500/95 ms; FOV of 216 × 256 mm^2^; matrix size of 108 × 128; resolution of 2 × 2 × 2 mm^3^; and 64 slices with no gap, covering the whole brain. Averages of 2 sets of diffusion-weighted images with phase-encoding directions of opposite polarity (left–right) were acquired to correct for susceptibility distortion. For each average of diffusion-weighted images, 4 images without diffusion weighting (*b* = 0 s/mm^2^) were also acquired with matching imaging parameters. The total diffusion MRI scan time was approximately 20 min. T1-weighted images were acquired with a 3D MPRAGE sequence (GRAPPA factor of 2) for all participants. The scan protocol, optimized at 3T, used a TR/TI/TE of 2600/900/3.02 ms, flip angle of 8°, volume of view of 224 × 256 × 176 mm^3^, matrix of 224 × 256 × 176, and resolution of 1 × 1 × 1 mm^3^. Total T1 scan time was approximately 4 min.

The resulting DTI data were processed using the FMRIB's Diffusion Toolbox (FDT) provided by FSL (FMRIB 4 Software Library) (Behrens et al., 2003). The FDT probabilistic tractography parameters were set to their default values (number of streamlines = 5000, maximum number of steps = 2000, loop check: set, curvature threshold = 0.2, step length = 0.5 mm, no distance bias correction).

We applied MANIA on 18 corticolimbic ROIs. All ROIs are in the left hemisphere and localized in Montreal Neurological Institute (MNI) standard space using the Automated Anatomical Labeling (AAL) (Jenkinson and Smith, 2001) of the WFU PickAtlas toolbox (Lancaster et al., 2000). The ROI acronym as well as the number of voxels in each ROI are shown in Table 1. The shape of these ROIs are not dilated and we adhere to the standard masks provided in the WFU PickAtlas toolbox. We chose these ROIs because they are known to play a significant role in various psychiatric disorders such as MDD, PTSD, OCD, anxiety and addiction (Mayberg, 1997; Seminowicz et al., 2004; Craddock et al., 2009; James et al., 2009). This sample of ROIs includes cortical, subcortical and limbic regions. Specifically, the cortical ROIs are BA6, BA9, BA10, BA40, BA46, BA47, the limbic are BA24, Th, BS, and the sub-cortical are BA11, BA25, BA32, Acb, Amg, Hp, Ht, Ins, Pc.

**Table 1:** **The 18 left hemisphere corticolimbic ROIs we consider in the case-study**.

**ROIs**	**Acronym**	**Number of voxels**
Premotor cortex	BA6	3131
Insula	Ins	1858
Ventromedial prefrontal cortex	BA10	1784
Inferior parietal cortex	BA40	1598
Dorsolateral prefrontal cortex	BA9	1422
Mid-brain and pons	BS	1406
Orbito-frontal cortex	BA11	1243
Thalamus	Th	1100
Hippocampus	Hp	932
Precuneus	Pc	861
Inferior prefrontal gyrus	BA47	851
Ventral anterior cingulate	BA32	721
Dorsal anterior cingulate cortex	BA24	593
Dorsolateral prefrontal cortex	BA46	574
Amygdala	Amg	220
Subcallosal cingulate	BA25	204
Nucleus accumbens	Acb	140
Hypothalamus	Ht	13

## Results

### Evaluation with the FiberCup dataset

**Figure 5:**
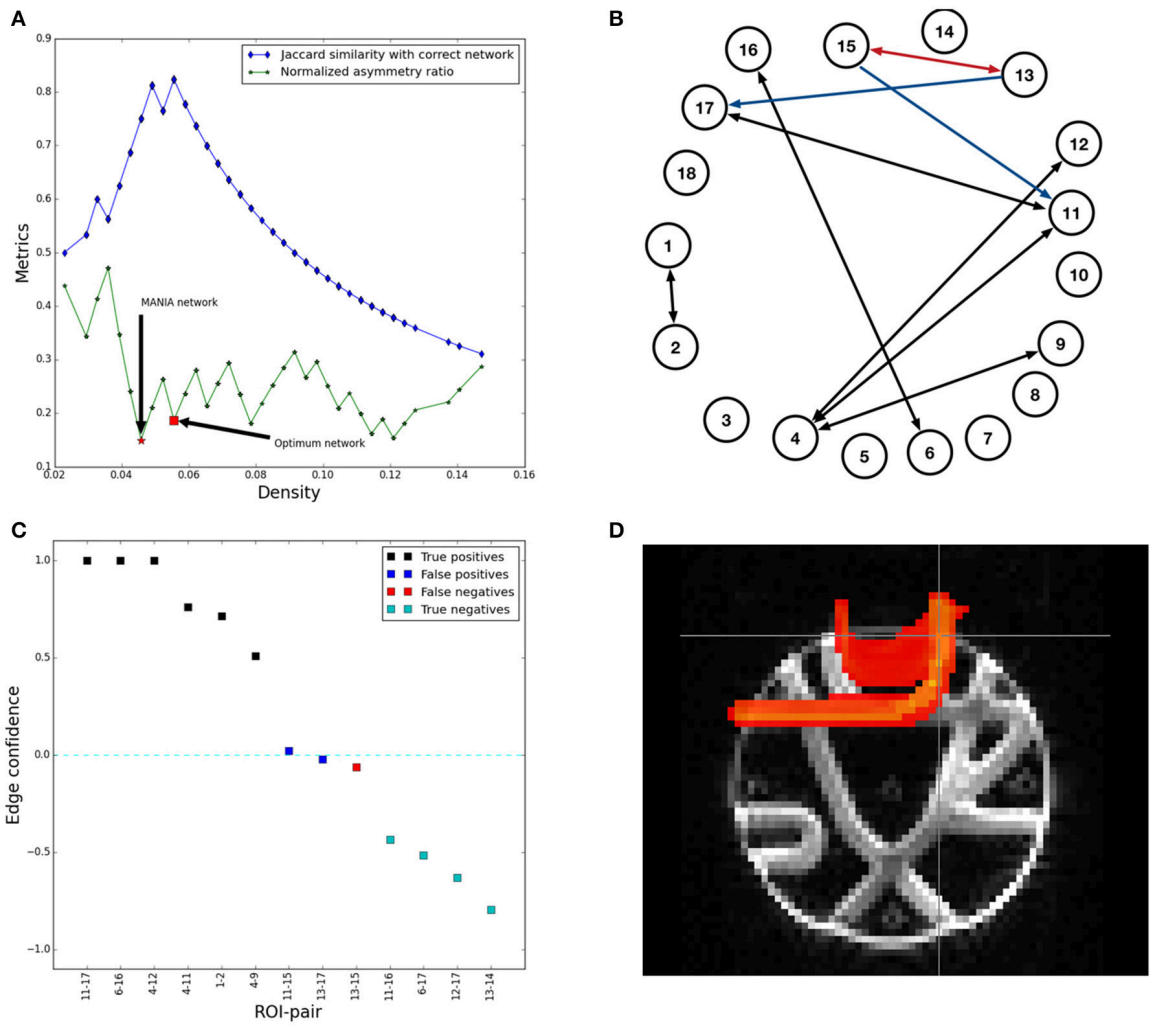
**Running MANIA on the FiberCup dataset. (A)** The normalized asymmetry metric and the network density that results from MANIA as well as from the optimal threshold. The Jaccard similarity with the correct, ground-truth network is also shown. **(B)** The network inferred by MANIA. **(C)** The thirteen ROI-pairs with the largest confidence metric. **(D)** The probability map generated by Probtrackx when seeded at the WGB of ROI-13.

Figure 5A shows that the MANIA threshold results in almost the same accuracy (Jaccard similarity within 5%) as the optimal threshold (see the Optimal Threshold-based Network Inference section). However, neither MANIA nor the optimal threshold are able to perfectly reconstruct the correct network. Figure 5B shows the network inferred by MANIA. There are two asymmetric false-positive edges (shown in blue) and one false-negative edge (shown in red). However, the confidence metric (shown in Figure 5C) of the three miss-classified edges is close to zero, while the confidence metric of the true-positives and true-negatives is significantly different than zero. Further, if we also perform post-symmetrization on MANIA's output, the false positive edge from ROI-13 to ROI-17 is removed because it has negative confidence.

Figure 5D explains the root-cause of the previous miss-classified edges. Seeding Probtrackx at ROI-13, most streamlines are incorrectly directed to ROI-17 (instead of ROI-15) because of the overlap with the fiber bundle between ROI-11 and ROI-17. The probability map generated by Probtrackx makes its highly unlikely that any threshold-based network inference method would be able to discover the right connection between ROI-13 and ROI-15.

### Evaluation with synthetic data

We evaluate MANIA based on computational experiments with synthetic data and random networks. The “ground-truth” networks *G* are constructed as follows. Suppose that *G* has *N* nodes and density ρ. We place $\left[\rho \frac{N\left(N-1\right)}{2}\right]$ undirected edges between randomly selected but distinct pairs of nodes. Note that *G* is symmetric by construction because the tractography process cannot infer the true polarity of the underlying neural fibers. Given *G*, we then create the tractography matrix *T* that represents the “noisy” fraction of streamlines between any pair of nodes, as shown in Equation (15). Note that the fraction of streamlines from a node X to a node Y is typically different than the fraction of streamlines from Y to X. In the following experiments, *N* is set to 50 nodes, and each experiment is repeated for 1000 networks *G*.

**Figure 6:**
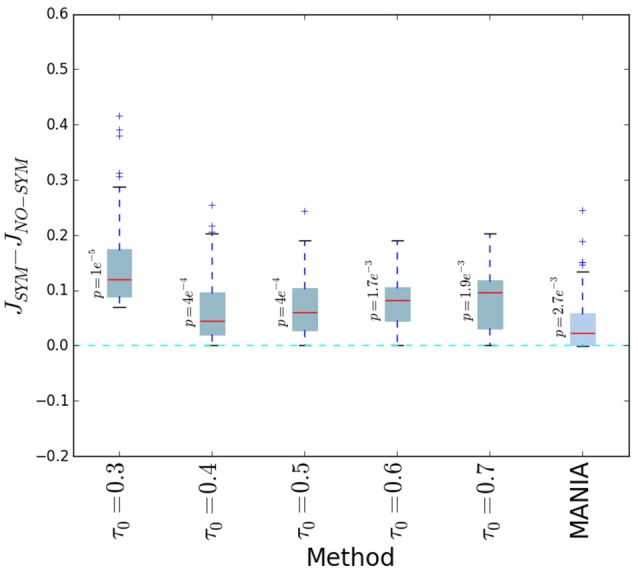
**The Jaccard similarity difference (Δ***J*** = ***J***_***SYM***_ − ***J***_***NO***−***SYM***_) with and without post-symmetrization for five threshold values and for MANIA**. Each box plot is generated from 1000 experiments; in each experiment we generate a random network with density between 0 and 1, while the noise parameters μ_1_ and μ_2_ are uniformly distributed between 0 and 0.3. The red line corresponds to the median, the box boundaries to the 25 and 75th percentiles, while the dashed lines show the 10 and 90th percentiles. In all cases, Δ*J* > 0 (one-sided Mann-Whitney *U*-test—*p*-values are shown next to each box plot) meaning that *post-symmetrization helps to improve the accuracy of network inference*.

We first examine the effect of post-symmetrization on the accuracy of both threshold-based network inference and MANIA. In the former, the connections are determined based on a given threshold τ_0_, as discussed in the Threshold-based Network Inference with Post-Symmetrization section. We denote the Jaccard similarity between the inferred network and the ground-truth network with *J*_*SYM*_ when post-symmetrization is performed, and with *J*_*NO*−*SYM*_ otherwise. Figure 6 shows the difference Δ*J* = *J*_*SYM*_ − *J*_*NO*−*SYM*_ for several choices of τ_0_ as well as for MANIA. Each box-plot is generated from 1000 experiments; in each experiment we generate a random network with density between 0 and 1, while the noise parameters μ_1_ and μ_2_ are uniformly distributed between 0 and 0.3. The red line corresponds to the median, the box boundaries correspond to the 25 and 75th percentiles, while the dashed lines show the 10 and 90th percentiles. In all cases, Δ*J* > 0 (one-sided Mann-Whitney *U*-test—*p*-values shown next to each box plot), meaning that *post-symmetrization helps to improve the accuracy of network inference*. This is true for both MANIA and threshold-based inference, even though the improvement is larger for the latter. Because of the positive effect of post-symmetrization, in the rest of the paper we apply it in all network inference experiments.

**Figure 7:**
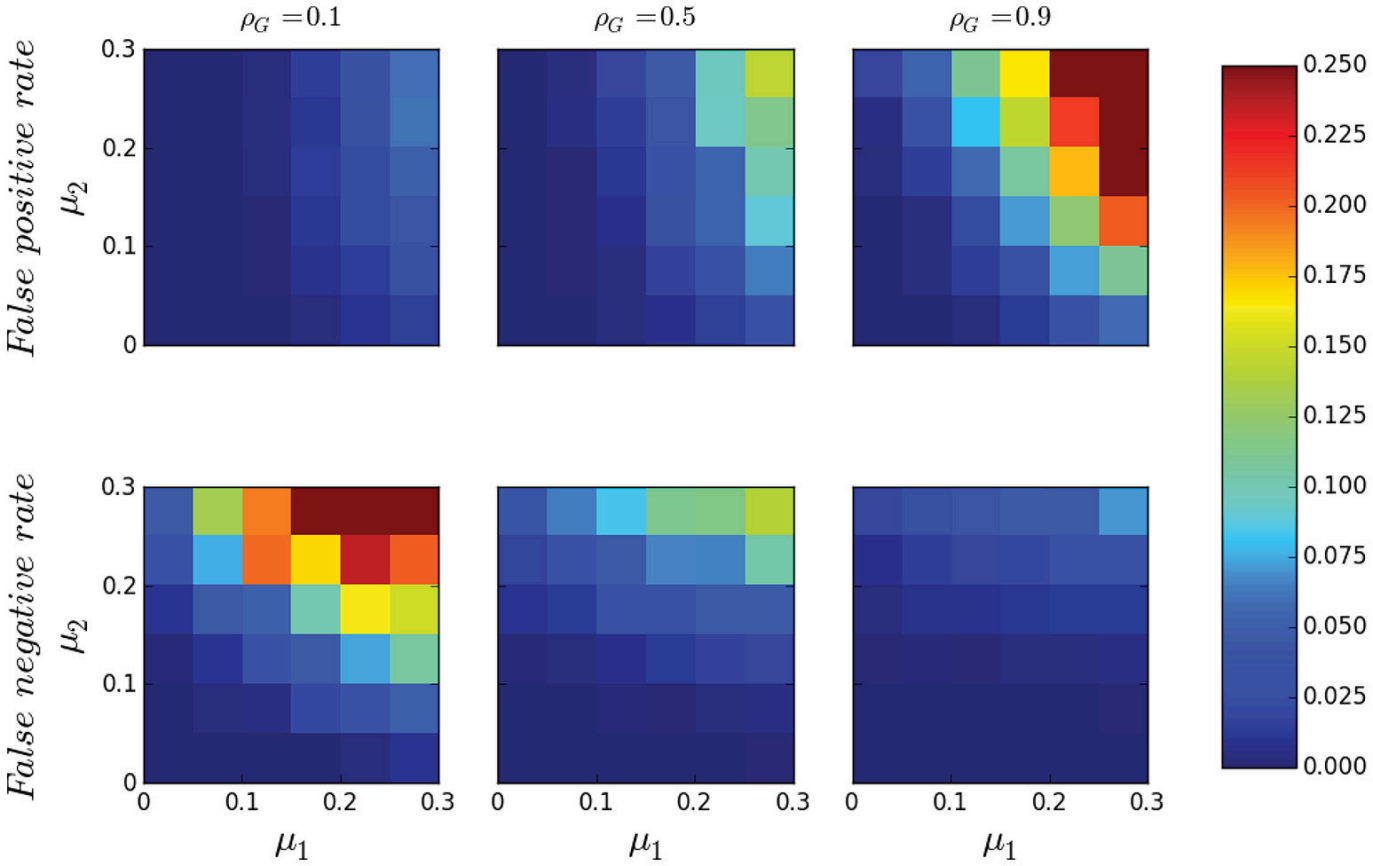
**False positive rate and false negative rate of MANIA as a function of μ_**1**_ and μ_**2**_ for sparse networks (ρ_***G***_ = 0.1), medium density networks (ρ_***G***_ = 0.5) and dense networks (ρ_***G***_ = 0.9)**. Each square is the average of 1000 independent simulations.

Figure 7 illustrates the performance of MANIA in the two-dimensional space defined by the noise parameters μ_1_ and μ_2_ for three values of the network density. Each square in these heat maps is the median across 1000 experiments. The false positive and false negative rates are close to 0 (less than 5%) in the lower-left half of each heat map (i.e., when μ_1_ + μ_2_ <0.3). For higher values of the noise intensity, the accuracy of MANIA depends on the density of the underlying network. In the case of sparse networks, MANIA also infers a sparse network and most errors are false negatives, i.e., MANIA does not detect some of the few existing edges. For dense networks, MANIA also infers a dense network and most errors are false positives, i.e., MANIA detects a few extra edges that do not actually exist. In mid-range densities, the errors are more balanced between false positives and false negatives. In all cases the maximum false positive (or negative) rate when μ_1_ = μ_2_ = 0.3 is less than 25%. Recall from Figure 4 that these noise intensity levels should be considered quite high in practice.

**Figure 8:**
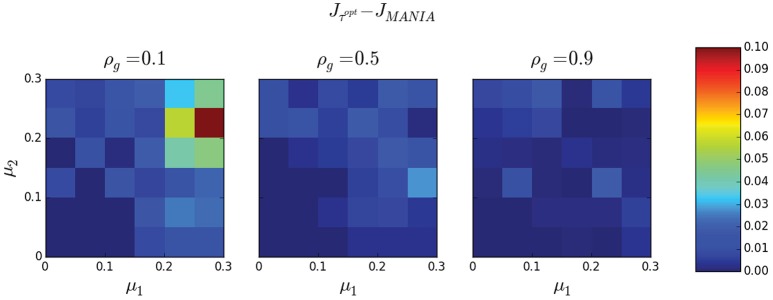
**The Jaccard similarity difference ($\Delta J=J_{\tau^{\text{opt}}}-J_{MANIA}$) between MANIA and the optimal threshold-based scheme as a function of μ_**1**_ and μ_**2**_ for sparse networks (ρ_***G***_ = 0.1), medium density networks (ρ_***G***_ = 0.5) and dense networks (ρ_***G***_ = 0.9)**. Each square is the average of 1000 independent simulations.

Figure 8 compares the MANIA-inferred network with the network that corresponds to the optimal threshold τ^*opt*^, as discussed in the Optimal Threshold-based Network Inference section. Specifically, the heat maps of Figure 8 compare the Jaccard similarity *J*_*MANIA*_ between MANIA and the ground-truth network, with the Jaccard similarity $J_{\tau^{opt}}$ between the optimal threshold based network and the ground-truth network. The accuracy of MANIA is typically close to that of the optimal threshold method. Even under the highest noise intensity we consider (μ_1_ = μ_2_ = 0.3), *J*_*MANIA*_ is only 10% lower than $J_{\tau^{opt}}$. These results suggest that MANIA selects automatically a connectivity threshold value that results in almost optimal accuracy, across all possible such threshold values.

**Figure 9:**
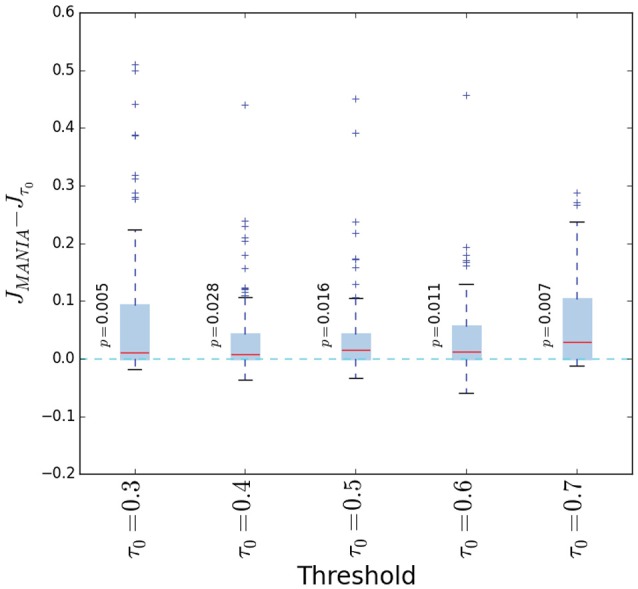
**The Jaccard similarity difference (Δ***J*** = ***J***_***MANIA***_ − ***J***_**τ**_**0**__) between MANIA and five given threshold values**. The accuracy comparisons are made after post-symmetrization. Each box plot is generated from 1000 experiments; in each experiment we generate a random network with density between 0 and 1, while the noise parameters μ_1_ and μ_2_ are uniformly distributed between 0 and 0.3. The red line corresponds to the median, the box boundaries to the 25 and 75th percentiles, while the dashed lines show the 10 and 90th percentiles. In all cases, Δ*J* > 0 (one-sided Mann-Whitney *U*-test—*p*-values are shown next to each box plot) meaning that *MANIA is more accurate than inferring the network based on a fixed threshold*.

Finally, Figure 9 compares MANIA with five given threshold values τ_0_. The comparison is in terms of the Jaccard similarity difference Δ*J* = *J*_*MANIA*_ − *J*_τ_0__. As in Figure 6, each box-plot is generated from 1000 experiments in which we vary the network density between 0 and 1, and the noise parameters μ_1_ and μ_2_ between 0 and 0.3. The median Δ*J* is always positive and the distribution of Δ*J* is skewed toward positive values (one-sided Mann-Whitney *U*-test—*p*-values shown next to each box plot), meaning that MANIA typically performs better than a fixed threshold scheme, independent of the selected threshold.

### Case-study: a rank-aggregated network between 18 ROIs

**Figure 10:**
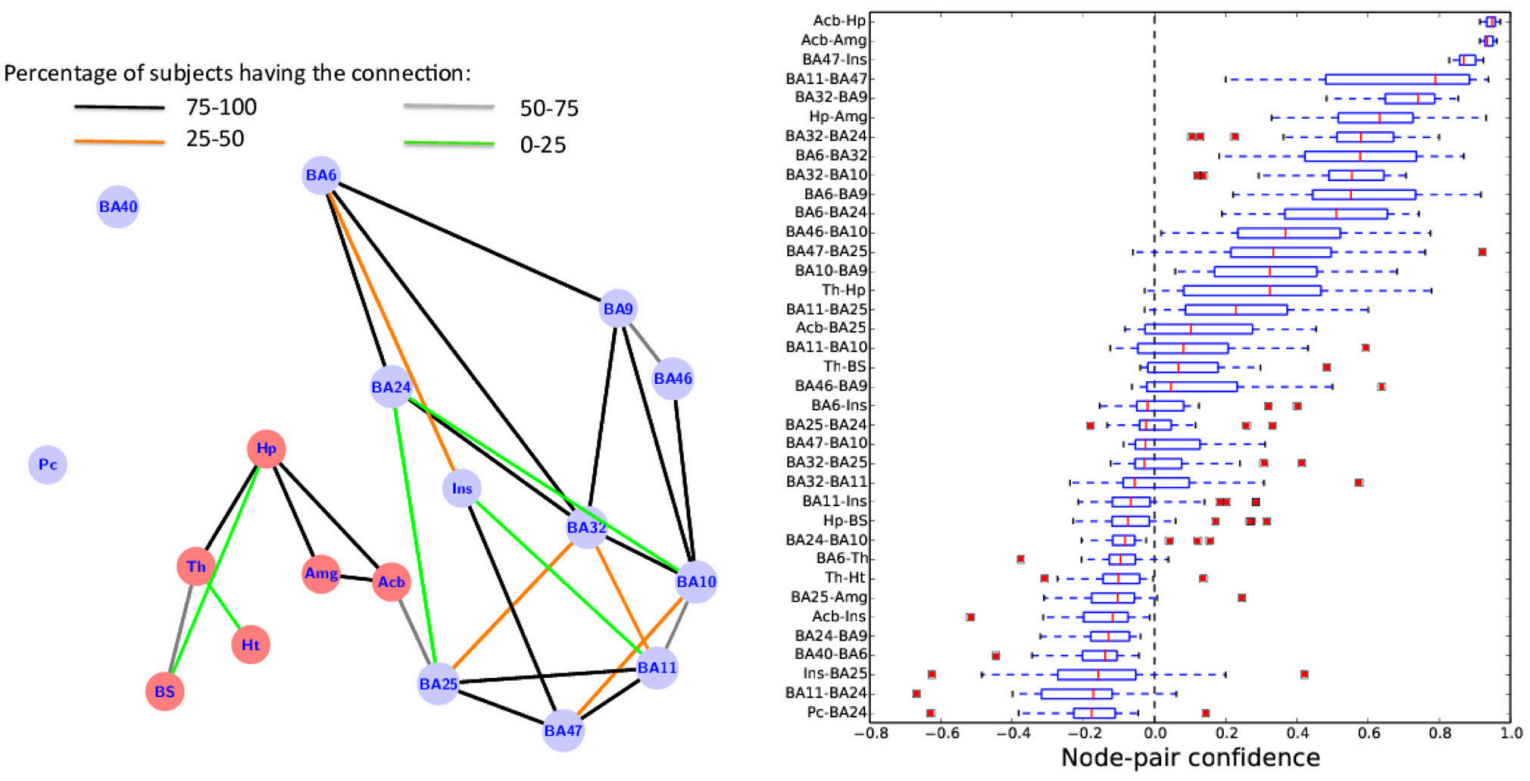
**(Left)** The rank-aggregated network, based on DTI data from 28 subjects, between the 18 ROIs in Table 1. Every edge in the connected component has been detected in both directions, i.e., MANIA identifies a completely symmetric network (no post-symmetrization is needed). The density of the connected component is 19%. The color of each edge represents the fraction of subjects that have the corresponding edge in their individual MANIA-based networks. **(Right)** Several percentiles of the node-pair confidence metric for each node-pair that appears connected in at least one of the 28 subjects.

We applied MANIA in the DTI data presented in DTI Data, Tractography Parameters, and Selected ROIs, based on the 18 ROIs listed in Table 1. A single rank-aggregated network is constructed, using the group analysis method of the Group Analysis section. Using MANIA, aggregating data from 28 subjects. The rank-aggregated network is shown in the left graph of Figure 10.

Two ROIs (Pc and BA40) appear to not be *directly* connected with the other 16 ROIs; of course there may be indirect connections through other ROIs that have not been included here (we return to this point in the Discussion section). Every edge in the connected component of Figure 10 has been detected in both directions, i.e., MANIA identifies a completely symmetric network in this case (i.e., no post-symmetrization is needed). The density of the connected component (16 ROIs) is 19%. The color of each edge in the subfigure represents the fraction of the 28 subjects that have the corresponding edge in their individual networks (constructed by MANIA).

We measured the “centrality” of each node in the rank-aggregated network, based on four centrality metrics (degree, closeness, betweenness, PageRank) (Newman, 2010). Different centrality metrics focus on different notions of importance. For instance, the degree centrality metric associates importance with the number of direct connections a node has; BA32 (Ventral anterior cingulate) has the largest number (six) of direct connections in this network (see Table 2). This may be because BA32 is spatially adjacent to both BA10 and BA25, and those ROIs are also of high degree. The betweenness centrality of a node X, on the other hand, focuses on the number of shortest paths between any pair of nodes that go through X; BA25 (subcallosal cingulate) is the most important node from this perspective because it serves as the “unique bridge” between the 6 red nodes at its left and the 9 blue nodes at its right. BA25 is also the most central node in terms of its average distance to all other nodes (closeness centrality).

**Table 2:** **Top-three nodes in rank-aggregated network based on four node-centrality metrics**.

**Centrality**	**Top-three nodes**		
Degree	BA32	BA10	BA25
Closeness	BA25	BA32	Acb
Betweenness	BA25	Acb	Hp
PageRank	BA10	BA32	BA11

Similarly, we measured the edge centrality of all connected node pairs. In terms of edge betweenness centrality, the connection between BA25 and the Nucleus Accumbens (Acb) is by far the most central in this network. It is interesting to note that this edge includes the segment of white matter that is the target of Deep Brain Stimulation (DBS) therapies for the treatment of MDD (Mayberg et al., 2005). In fact, the DBS target is typically the point at which the fibers between (BA25-Acb), (BA25-BA32), and (BA25-BA24) intersect.

Figure 10 also shows some percentiles of the per-subject node-pair confidence metric (median, 25–75th percentiles, 10–90th percentiles, and outliers) for each node-pair that appears connected in at least one of the 28 subjects. The connections between the following node pairs appear in all subjects and have the highest confidence: Hp-Acb, Amg-Acb, BA47-Ins. On the other hand, the following connections appear only in some subjects and their confidence metric varies around zero: Th-BS, BA46-BA9, BA6-Ins. Some connections that appear in 1–2 subjects but have very low confidence are: Pc-BA24, BA11-BA24, Ins-BA25, BA40-BA6.

## Discussion

Thomas et al. have recently shown that inferring long-range anatomical connections between gray matter ROIs from DWI data can be significantly inaccurate (Thomas et al., 2014). The authors also note that “*(probabilistic tractography methods) are less susceptive to changes in the composition of an ROI but only if an optimized threshold can be derived and used.”* More recently Reveley et al. (2015) have investigated the key reasons behind the negative results of Thomas et al. (2014). They showed that the dense system of white matter fibers residing just under the cortical sheet poses severe challenges for long-range tractography, concluding that it is “*extremely difficult to determine precisely where small axonal tracts join and leave larger white matter fasciculi.”*

In light of the previous results, it is clear that there is a need for new network inference methods. We believe that MANIA is moving in the right direction for the following reasons:

- The results of Thomas et al. (2014) suggest that probabilistic tractography can be more accurate than deterministic tractography in terms of sensitivity and specificity as long as its parameters are appropriately optimized. MANIA is indeed mostly applicable to probabilistic tractography, and its main focus is how to “self-configure” its connectivity threshold τ in an optimized manner, relying on what we expect to be true about the structure of the resulting solution (namely, a symmetric network).
- The results of Reveley et al. (2015) suggest that it is risky to dilate the given ROIs, which are typically mostly gray matter, so that they also include some white matter voxels. Those voxels may be part of the white matter fiber systems that reside just under the cortical sheet. In other words, if our goal is to understand the connectivity between gray matter ROIs, we should not use tractography seeds that reside in white matter; instead, we need to seed from gray matter even if the diffusion signal is much weaker there. So, we need to expect that some connections may appear as asymmetric, which is what MANIA anticipates.
- The results of Reveley et al. (2015) can be interpreted as follows: because it is hard for any tractography method to accurately cross the WGB, especially in the case of cortical ROIs, a network inference method should be able to deal somehow with erroneous measurements about specific connections. In other words, just because tractography failed to cross the WGB going from seed X to target Y does *not* mean that we should conclude that X and Y are not connected. And so, given that the input data about individual connections is quite noisy, we need to examine if there is any additional “hidden structure” in the inference problem that we can exploit. If this is case, we can then look for a solution that satisfies the constraints of that additional structure. In MANIA, this “hidden structure” in the inference problem is that the resulting network should be as symmetric as possible.
- Recently, Zalesky et al. (2016) argued that inferring brain networks with both high specificity and sensitivity is beyond the capabilities of current diffusion-weighted MRI, fiber modeling and tractography methods. Further, they showed that specificity is at least twice as important as sensitivity when estimating certain topological properties of brain networks (clustering, efficiency, modularity). As shown in the Evaluation with Synthetic Data section, MANIA tends to emphasize specificity instead of sensitivity (i.e., most errors are false negatives) under sparse connectivity, such as the network between distinct brain modules.

Of course, we do *not* claim that MANIA addresses every concern about tractography-based network inference. On the contrary, there are more open issues that need to be addressed. Two of them are further discussed next.

Even if the thresholding problem is adequately addressed with MANIA, there is another important problem in structural network inference: the *distance bias* of the tractography process (Donahue et al., 2016). It is harder to discover connections between distal regions due to the accumulation of uncertainty in long streamlines, causing false negatives for long-range connections (Li et al., 2012a,b). Additionally, it is more likely to incorrectly detect connections between proximal regions, especially in the presence of crossing or turning fibers, causing false positives. The FDT toolbox provides a “distance correction” option by multiplying the number of streamlines that cross a voxel by the average length of those streamlines^4^—there is no evidence however that this simple form of distance correction is able to improve significantly the accuracy of the network inference process. More sophisticated methods exist however (Morris et al., 2008; Donahue et al., 2016). The method by Morris et al. compares the tractography-generated connectivity probabilities with a null model that gives the corresponding connectivity probabilities with a random tracking process that is dominated by the same distance effects. The method by Donahue et al. regresses out the distance bias from the inter-ROIs connection weights using a log-linear model. We view distance correction methods as an independent processing step that can be applied prior to applying MANIA. For instance, the method by Morris et al. first creates a “null frequency of connection map,” it then filters the “experimental frequency of connection map” that is produced by a probabilistic tractography tool, resulting in the so-called “significance of connection map” (which is supposed to have fewer false positives). MANIA can be then applied on the latter, rather than on the experimental frequency of connection map. It is still not clear if the Morris distance correction method is sufficient to completely address the distance correction bias (Taljan et al., 2011), or whether the method of Donahue et al. is sufficient to completely address the distance bias for cortico-subcortical connections (van den Heuvel et al., 2015). Nevertheless, we anticipate that the combination of MANIA with a distance correction method will improve the accuracy of the resulting networks.

MANIA does not produce a weighted network in which a strength metric is associated with each edge. It provides however a confidence metric for the presense or absense of each possible connection. Interpreting streamline counts in probabilistic tractography as an indication of connection strength is debatable (Jones et al., 2013). It is not yet clear how to differentiate between weak yet probable connections vs. strong but unlikely connections (Zalesky et al., 2016). Nevertheless, a weighted approach like that of Donahue et al. returns an almost fully connected network with weights that span several order of magnitudes (Donahue et al., 2016). In order to achieve good sensitivity and specificity, there is again the need to threshold this weighted connectome (see Figure 4D of the paper by Donahue et al.). For example, in Azadbakht et al. (2015) the authors achieve 74% accuracy but only if the optimum threshold is used. MANIA can address this thresholding problem by first pruning the tractography results to get a set of unweighted but likely connections. Then, one can define the desired weighting scheme for this set, e.g., through fractional scaling (see Donahue et al., 2016).

A network representation consists of both nodes and edges. The clinical and research value of representing a brain as a network depends critically on the selected nodes and on the exact boundaries of the corresponding ROIs (Zalesky et al., 2010). MANIA assumes that the set of given nodes is sufficiently specified, and that their spatial boundaries are accurately defined. In practice, this step of the network inference process is always an “inexact science” given that the functional role of any given ROI is at best only partially understood and the anatomical boundary of each ROI is subject-dependent (Tzourio-Mazoyer et al., 2002).

A voxel-level analysis (van den Heuvel et al., 2008) avoids the selection of functionally specific ROIs but it makes it harder to associate the topological properties of the observed network, which now consists of many thousands of nodes, to any known brain circuits and their function. Again, we view this important issue as orthogonal to MANIA: improved brain parcellation methods, such as data-driven parcellations (Power et al., 2011; Yeo et al., 2011; Glasser et al., 2016b) and decreasing voxel sizes can be used jointly with MANIA to identify structural networks that are consistent, or that can explain well, the observed spatio-temporal correlations in resting-state or task-based fMRI analyses. This coupled exploitation of fMRI and diffusion MRI data has provided valuable insights about the underlying anatomy of the brain structures that result in the Default Mode Network (Honey et al., 2009), and they can become more common now that the HCP project provides both functional and diffusion data for hundreds of subjects (Van Essen et al., 2013).

In our 18-ROI case-study, summarized in Figure 10, the use of mostly large ROIs that do not necessarily correspond to distinct functional units, together with the distance bias of the tractography process, may account for the lack of certain expected connections. Two such expected connections are between Pc and BA40 (Greicius et al., 2009), and between BA9 and BA40 (Petrides and Pandya, 2007); the latter is a long-distance connection. Additionally, large cortical ROIs such as BA9 and BA40 are only imprecisely defined, which may also explain the absence of some of their connections. The limbic and subcortical ROIs, on ther other hand, are more precisely defined and their connections are mostly running over shorter distances.

These findings suggest that MANIA should be evaluated in the future jointly with, first, advanced distance correction methods, and second, with either more precisely defined ROIs or on a whole-brain parcellation template.

## Author contributions

KS and CD designed research, KS, SB, DG, HM, and CD performed research, KS and SB analyzed data, and KS, HM, and CD wrote paper.

### Conflict of interest statement

The authors declare that the research was conducted in the absence of any commercial or financial relationships that could be construed as a potential conflict of interest.

## Footnotes

^2^In graph theory, an *N* × *N* adjacency matrix represents a directed and unweighted graph with *N* nodes as follows: if there is an edge from node i to node j the (i, j) element of the adjacency matrix is 1; otherwise it is 0. The graph (and the corresponding adjacency matrix) are referred to as “weighted” if each edge is associated with a weight, which typically represents the strength of the edge.

^3^Since we set the number of streamlines to 5000, the minimum resolution is $\tau =\frac{1}{5000}=0.0002$.

^4^http://fsl.fmrib.ox.ac.uk/fsl/fslwiki/FDT/UserGuide.

## Abbreviations

AD: Addictive Disorder
DTI: Diffusion Tensor Imaging
FACT: Fiber Assignment by Continous Tracking
FDT: FMRIB's Diffusion Toolbox
fMRI: functional Magnetic Resonance Imaging
FMRIB: Oxford centre for Functional Magnetic Resonance Imaging of the Brain
fODF-PROBA: Fiber Orientation Distribution Function-Probabilistic
FSL: FMRIB Software Library
GAD: Generalized Anxiety Disorder
HCP: Human Connectome Project
MANIA: Minimum Asymmetry Network Inference Algorithm
MDD: Major Depressive Disorder
MNI: Montreal Neurological Institute
MRI: Magnetic Resonance Imaging
OCD: Obsessive Compulsive Disorder
PiCo: Probabilistic Index of Connectivity
PTSD: Post-Traumatic Stress Disorder
ROI: Region Of Interest
WGB: White matter/Gray matter Boundary.
